# Supplementary material for: Acute and Subchronic Toxicity Studies of Aristolochic Acid A in Tianfu Broilers
Source: Animals (Basel). 2021 May 27;11(6):1556. doi: 10.3390/ani11061556 (PMC8228413; doi:10.3390/ani11061556)

Supplementary figure S1. The flow cytometry quadrant diagrams of ROS levels in kidney. a: CG group, b: LAG group, c: MAG group, d: HAG group.

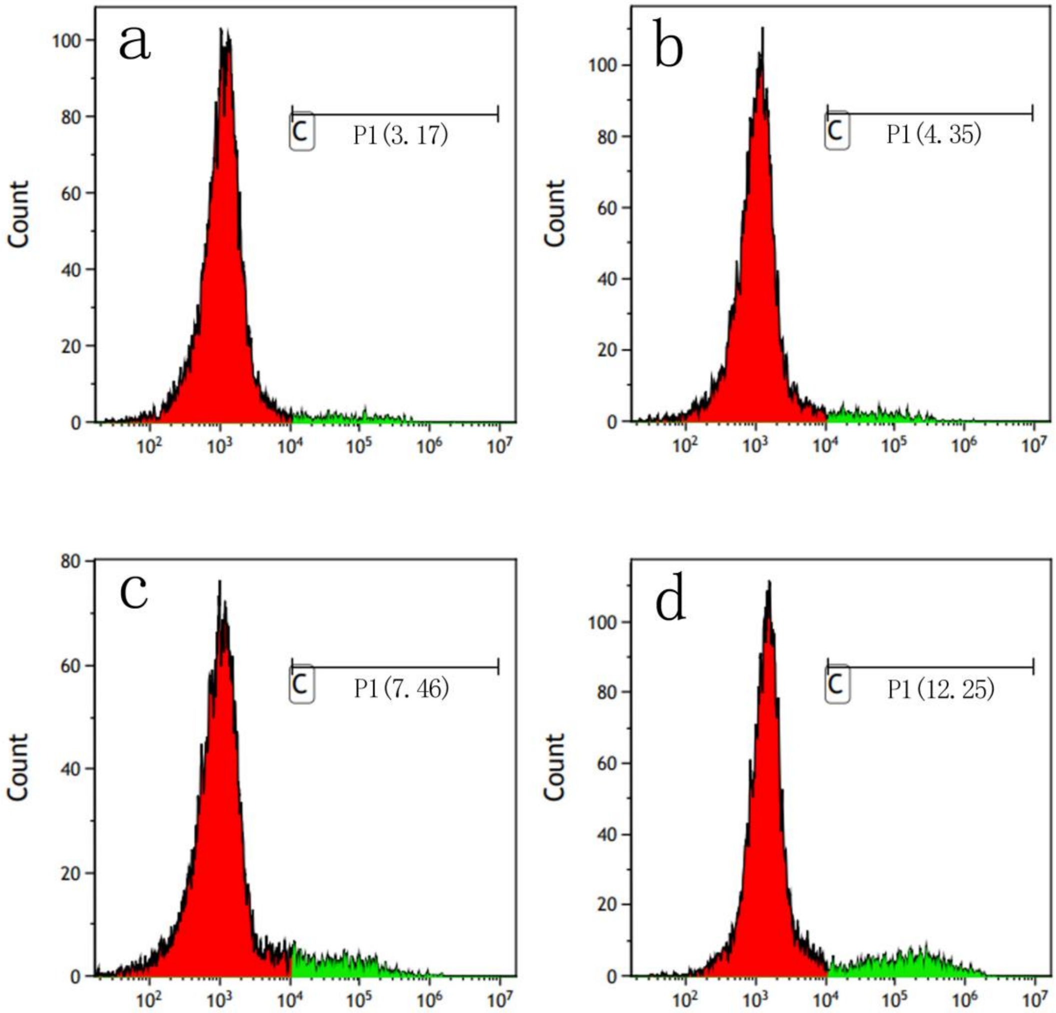

Supplementary figure S2. The flow cytometry quadrant diagrams of apoptosis in kidney. a: CG group, b: LAG group, c: MAG group, d: HAG group.

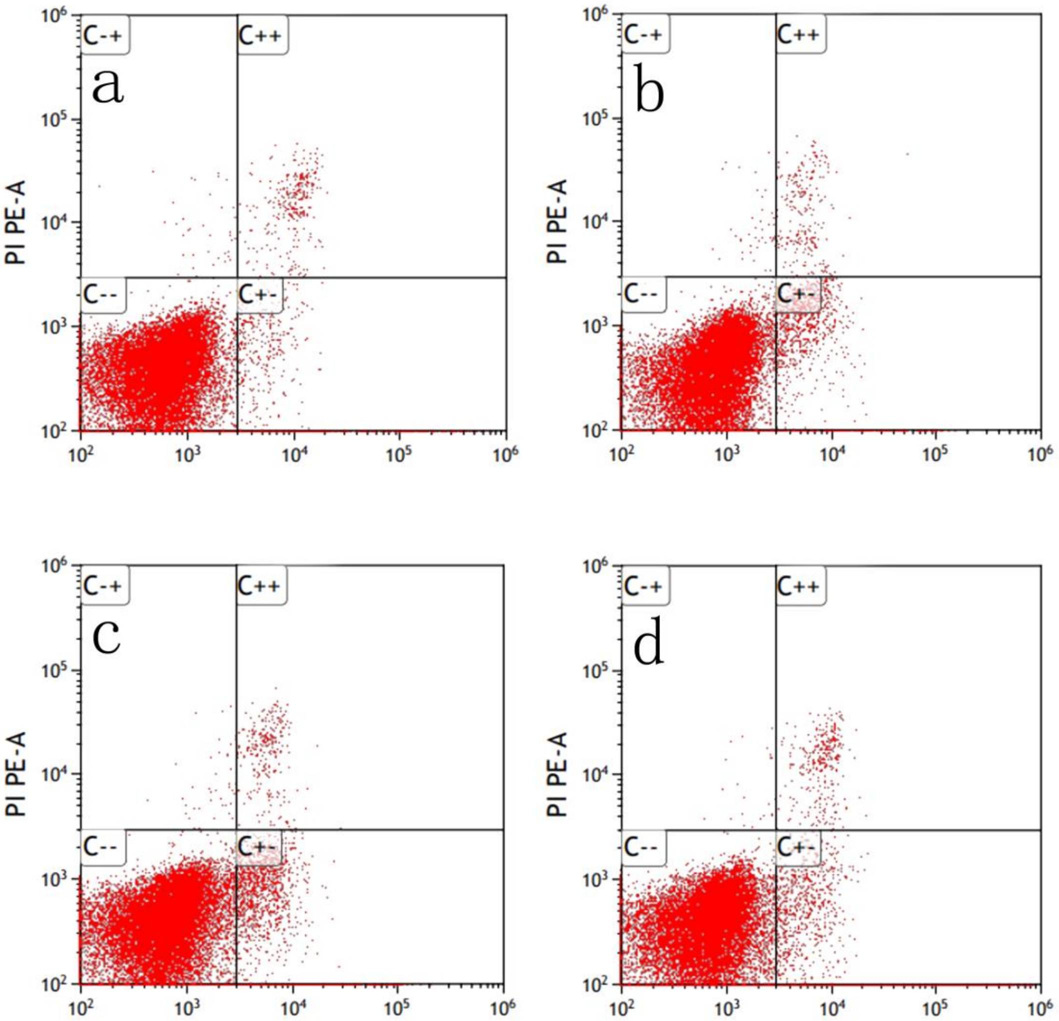

Supplementary figure S3. The flow cytometry quadrant diagrams of mitochondrial depolarization ratio in kidney. a: CG group, b: LAG group, c: MAG group, d: HAG group.

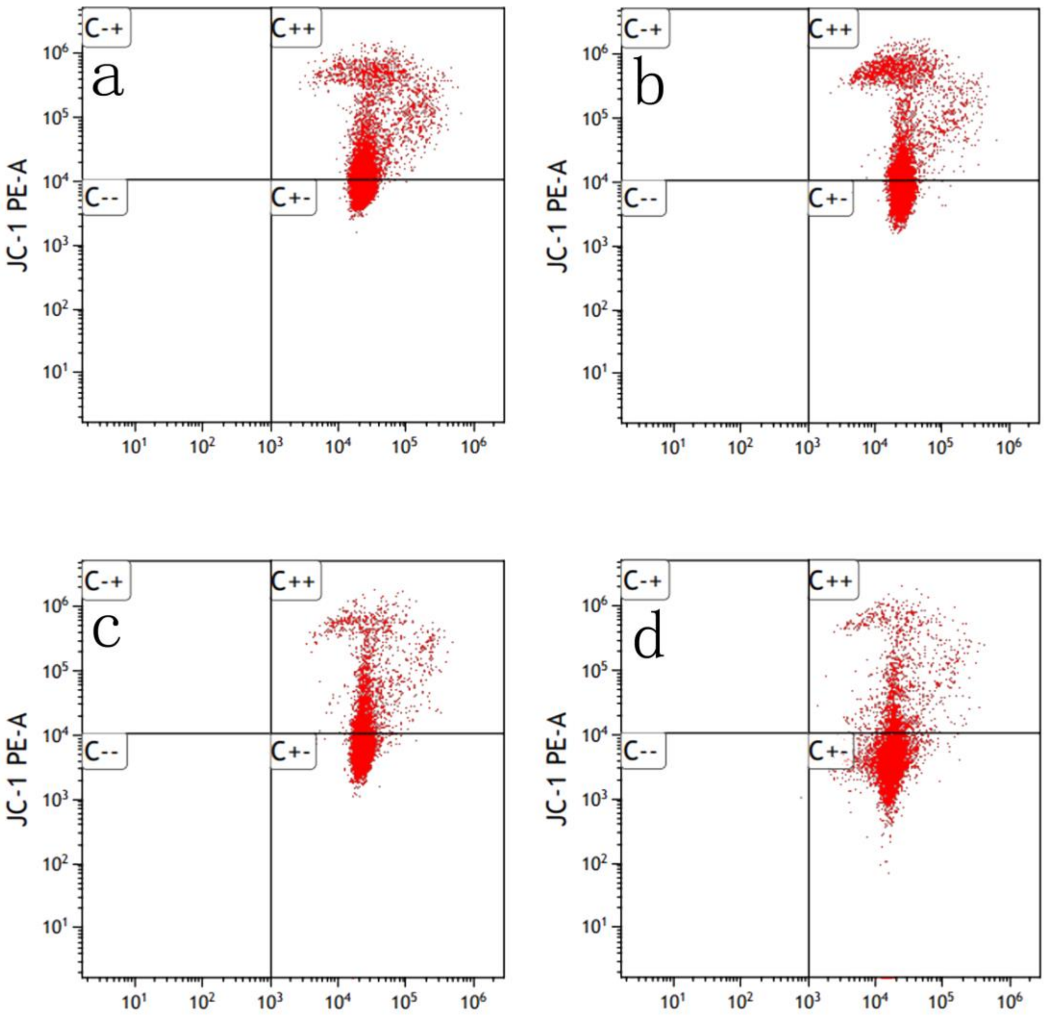

Supplement: Supplementary file 1 [file animals-11-01556-s001.zip › supplementary figures.pdf]
